# An alternative splicing signature in human Crohn’s disease

**DOI:** 10.1186/s12876-021-02001-2

**Published:** 2021-11-08

**Authors:** Daowei Li, Yuanzi Liang, Jia Lu, Yue Tan

**Affiliations:** 1 Department of Radiology, The People’s Hospital of China Medical University and The People’s Hospital of Liaoning Province, No. 33, Wenyi Road, Shenhe District, Shenyang, 110016 China; 2 Department of Gastroenterology, Shengjing Hospital of China Medical University, 39 Huaxiang Road, Tiexi District, Shenyang, 110022 China

**Keywords:** Alternative splicing, Crohn’s disease, Post-transcriptional regulation

## Abstract

**Background:**

Although hundreds of risk loci for Crohn’s disease (CD) have been identified, the underlying pathogenesis of CD remains unclear. Recently, evidence has shown that aberrant gene expression in colon tissues of CD patients is associated with the progression of CD. We reasoned that post-transcriptional regulation, especially alternative splicing (AS), may also play important roles in the pathogenesis of CD.

**Methods:**

We re-analyzed public mRNA-seq data from the NCBI GEO dataset (GSE66207) and identified approximately 3000 unique AS events in CD patients compared to healthy controls.

**Results:**

“Lysine degradation” and “Sphingolipid metabolism” were the two most enriched AS events in CD patients. In a validation study, we also sequenced eight subjects and demonstrated that key genes that were previously linked to CD, such as *IRF1* and *STAT3*, also had significant AS events in CD.

**Conclusion:**

Our study provided a landscape of AS events in CD, especially as the first study focused on a Chinese cohort. Our data suggest that dysregulation of AS may be a new mechanism that contributes to the pathogenesis of CD.

**Supplementary Information:**

The online version contains supplementary material available at 10.1186/s12876-021-02001-2.

## Background

Crohn’s disease (CD), an inflammatory bowel disease (IBD), is thought to be caused by dysregulated mucosal immune responses to the gut flora in genetically susceptible patients [1]. To date, more than 200 risk loci for IBD have been identified, which are also enriched in multigenic regulatory modules [2, 3]. Although genome-wide association studies and subsequent meta-analyses could explain the underlying pathophysiology of CD to some extent, the pathogenesis of CD remains unclear. Recent studies have shown that dysregulated gene expression in the gut mucosa of CD patients may determine the initiation and progression of CD [4]. A subsequent study has also showed that there is a large difference in the gene expression of inflamed and non-inflamed intestinal mucosa from CD patients when compared the gene expression of healthy mucosa from controls [5]. Moreover, a study that focused on integrated analysis of micro-RNAs (miRNA) and mRNA expression have demonstrated that miRNAs also play critical roles in regulating gene expression in CD patients [6]. Some of these circulating miRNAs are also thought to be potential prognostic markers in CD patients [7]. These data suggest that transcriptional and post-transcriptional regulation may play important roles in determining the etiology of CD.

Protein diversity determines the complexity of the eukaryotic cellular processes. Alternative splicing (AS) of the precursor mRNA is one of the essential mechanisms for increasing protein diversity and regulating intricate protein-RNA networks [8, 9]. Nearly 95% of the total human multi-exon genes are involved in AS events [10]. Evidence indicate that AS plays crucial roles in not only oncogenic processes, including proliferation, apoptosis, hypoxia, angiogenesis, immune escape, and metastasis [11, 12], but also in basic developmental processes and tissue identity [13]. A pioneering study, which have focused on the AS of pre-mRNA in IBDs, have identified 47 splicing factors and 33 intron retention events that are dysregulated in the mucosal tissue of patients [14]. However, due to the lack of next-generation sequencing technology, only 149 splicing factors and 145 intron retention events were screened in that pioneering study.

We reasoned that dysregulated AS could contribute to the pathogenesis of CD. Thus, we obtained public mRNA-seq data from the NCBI GEO dataset (GSE66207) [6], which included 33 colon tissue mRNA-seq data (20 CD and 13 healthy controls). Using Mixture-of-Isoforms (MISO) and related AS event analysis software [15], we identified approximately 3000 significant AS events in CD patients compared in controls. A total of 117 biological pathways were significantly enriched for these AS events, some of which were highly related to inflammation-related responses. interferon regulatory factor 1 (*IRF1)*, signal transducer and activator of transcription 3 (*STAT3)*, and monoamine oxidase A *(MAOA)* were identified as key genes involved in these AS events. In a validation study, we also identified that 64% of genes of significant AS events that overlapped with genes present in results from the public dataset, indicating a comprehensive role of AS in CD. We believe our results may shed light on the mechanism of post-transcriptional regulation of CD progression.

## Methods

### Sample collection

A total of four patients with CD from the Shengjing Hospital of China Medical University, Shenyang, China, were recruited between June 2020 and September 2020 and served as the experimental group. All eligible patients had an established diagnosis of CD based on endoscopic and histologic assessments. Colonic biopsy specimens were taken from the rectum, ulcer margin of the sigmoid colon, and inflamed portions. Four patients with normal distal colon confirmed by surgical pathology served as the control group. The study was approved by the institutional review board of the Shengjing Hospital of China Medical University, and informed consent was obtained from each patient.

### Data preparation

RNA-seq data were obtained from the National Center for Biotechnology Information (NCBI) Gene Expression Omnibus (GEO) dataset (GSE66207) [6]. Sequencing was performed via ILLUMINA (Illumina HiSeq 2500) mean run: 26.98 M spots. The mean size for the sequencing file includes 2.58G bases according to the SRX886282 project detail. This dataset included 20 Crohn’s disease patients and 13 normal colon tissues’ raw sequence data and mRNA expression data (patient information is included in Table 1). Fastq files were aligned on the human Hg19 genome using STAR-2.7.1a [16] (read length = 150, average read depth = 50). The indexed.bam files were generated by Samtools (1.10) [17] for the next step of the analysis. Raw files for validation of the experiment were prepared using the same method.

**Table 1:** Clinical Characteristics of the Patients (A total of 4 patients with CD were included between June 2020 and September 2020

	Patient1	Patient2	Patient3	Patient4
Age (years)	16	22	30	23
Gender	Male	Female	Female	Male
Course of disease (years)	1.5	0.5	4	3
Age at diagnosis	15	22	29	21
Disease phenotype	B3	B1	B1p	B2p
Disease location	L3	L3	L3	L2
CDAI	160	170	410	240
Medication	Anti-TNF-a therapy	5-ASA and Anti-TNF-a therapy	Anti-TNF-a therapy	Anti-TNF-a therapy and corticosteroids

The clinical characteristics of all patients are shown in Table 1. There were no significant differences with regard to age and sex between the experimental and control groups (*P* > 0.05).)

Locations: L1 = terminal ileum; L2 = colon; L3 = ileocolon. Behaviors: B1 = nonstricturing; B2 = stricturing, B3 = penetrating; P = perianal disease

ASA, Aminosalicylic acid

### Identification of significant AS events

AS events were identified and quantified using MISO version 0.5.4 [15] for 33 samples. A read length of 48 was applied, and other parameters were kept as default in MISO. The level of AS events was defined as the percent spliced in (PSI). To obtain differentially expressed AS events between Crohn’s disease patients and normal samples, we applied the Wilcoxon test on all AS events with at least three patients and one normal sample. Significant events were defined as those with *P* values < 0.05. The same method was applied in the transferability study. The AS events functional impact analysis was performed as significant events imported into the ASpedia database (http://combio.snu.ac.kr/aspedia/). Inputs of ASpedia analysis were converted from the MISO results.

### Pathway analysis

Pathway analysis of the splicing and data was performed using Enrichr [18, 19] with the Kyoto Encyclopedia of Genes and Genomes (KEGG) pathway library [20]. Only pathways with a *P* value < 0.05 were considered as related pathways. WebGestalt [21], SUMER [22] and Cytoscape (3.8.0) [23] were applied to the pathway analysis of the transferability study section. Overrepresentation analysis and gene ontology biological processes were applied as enrichment methods and functional databases, respectively and were conducted using WebGestalt. The enriched category with gene sizes less than 5-kb and a false discovery rate above 0.05 was removed from the WebGestalt results. Results of different AS types were input into SUMER to obtain the pathway network. Finally, Cytoscape (3.8.0) was applied to modify the color and text size of the network from SUMER.

### Visualization

A Sankey diagram was generated using Plotly in Python. UpSet plots were generated by ComplexHeatmap [24] in R (4.1.0). Sashimi plots were generated using Integrative Genomics Viewer (version 2.8.3) [25]. To avoid random error, we randomly selected and merged RNA-seq alignment data from three patients with Crohn’s disease and three normal samples. Other graphs are plotted using the ggplot in R.

### RNA-seq expression analysis

Pair-ended RNA-seq libraries for the transferability study were prepared using the NEBNext Ultra RNA with a Poly-A selection kit and were sequenced on an Illumina Hi-Seq 4000 (Genergy, Shanghai, China). Kallisto software was subsequently used to quantify RNA-seq raw counts based on a pseudoalignment algorithm [26] (read length = 200, default; mean read depth = 50). Differential gene expression was determined with log2foldchange > 1.5 and *P* < 0.05, genes with > 1 count per million. Any gene with a *P* value greater than the false discovery rate, after Benjamini–Hochberg correction for multi-testing, was deemed significantly differentially expressed under the test conditions as compared to the controls.

### Quantitative real-time PCR (RT-qPCR) analysis

Colon tissues from four control and four CD patients were used for qPCR assays. Total RNA was extracted from cells using TRIzol reagent (BioTeke, Beijing, China) and then reverse-transcribed using the first-strand cDNA synthesis kit (TSK302S, RT6 cDNA Synthesis Kit Ver 2). Reverse-transcribed products were used as templates for qPCR using the 2 × T5 Fast qPCR Mix (SYBR Green I). The primers used for the different target genes were as follows: *STAT3* (exon1)-F: GGCGAGGATTGGCTGAAGGG; *STAT3* (exon1)-R: CAGGCCGAAGGGCCTCTC; *STAT3* (lengthened 3’UTR)-F: tttctggaagttaaagtagatacagca; *STAT3* (lengthened 3’UTR)-R: ggccactgcattcaaattcc; *IRF1*(exon1)- F: GGCAGAGCTCGCCACTCCTTAGTC; *IRF1*(exon1)-R: AGGCAGAGGTTGCCGGGTT; *IRF1* (lengthened 3’UTR)-F: acatgtggctagtgccagtg; *IRF1* (lengthened 3’UTR)-R: cacagatgctgctccaaaaa.

## Results

### Splicing types

**Fig. 1:**
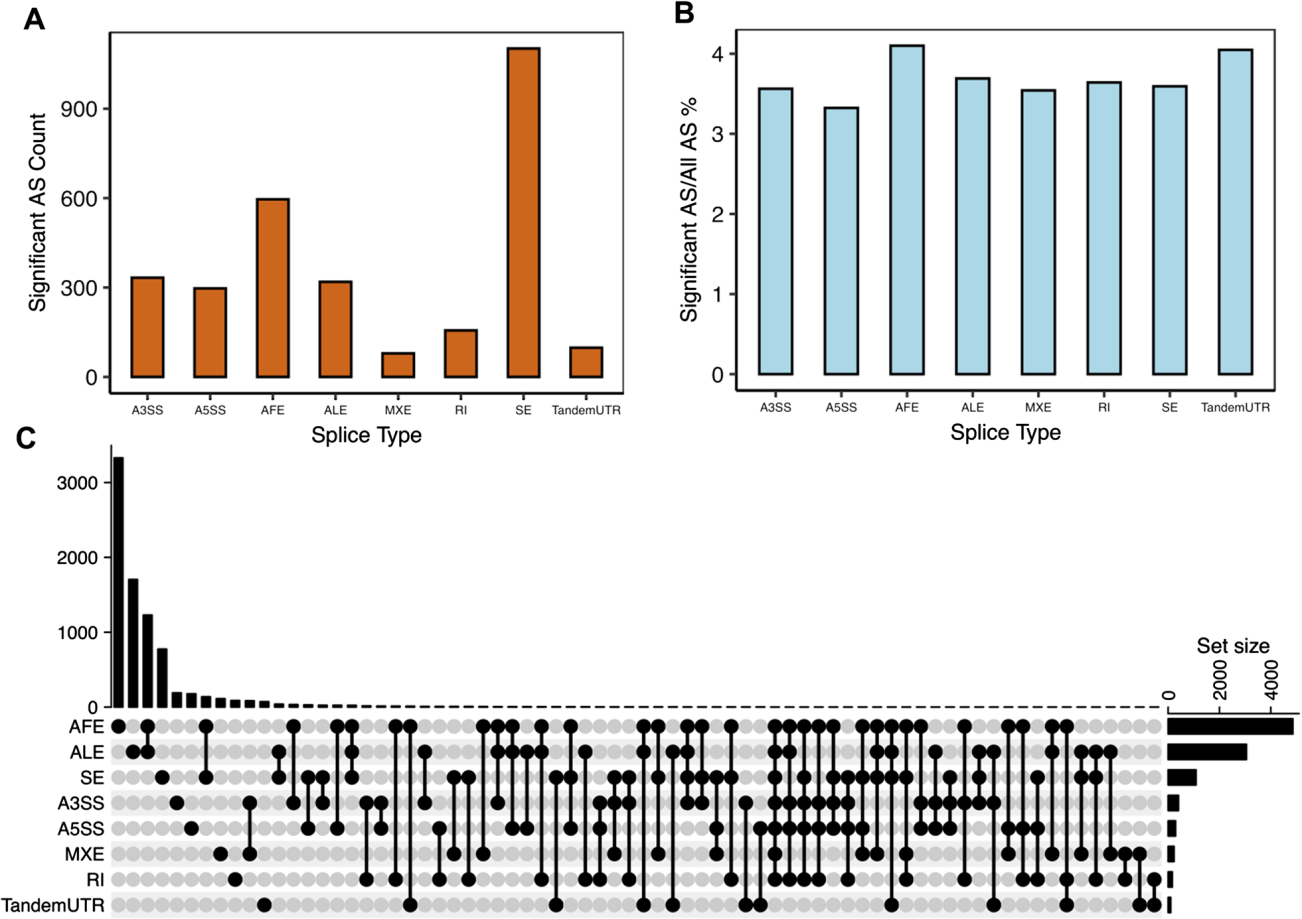
Alternative splicing identification in Crohn’ disease (CD) patients and normal colon samples. **a** Significant alternate splicing (AS) event counts in different AS types between CD patients and normal colon samples. **b** The percentage of significant AS events over all AS events in different AS types. **c** Distribution of AS types and intersections between gene symbols of 2980 related AS events

In the study of identifying AS events in the data from GSE66207, we found eight types of AS events, including alternative 3’ splice site (A3SS), alternative 5’splice site, alternative first exon (AFE), alternative last exon, mutually exclusive exons (MXE), retrained introns (RI), skipped exon (SE), and tandem 3’ UTR. A total of 2980 significant AS events were discovered in 20 patients with Crohn’s disease (Fig. 1a) (Additional file 1: Table S1). Among these types, SE had the most significant AS events, but the percentage of the total number of SE events was low. A total of 4.1% of AFE events and 4% of tandem 3’UTR events were recognized as significant events, indicating a higher level compared to other events (Fig. 1b). The distribution and intersection of gene symbols among the eight event types for 2980 significantly different AS events are shown in Fig. 1c.

### Pathway analysis

**Fig. 2:**
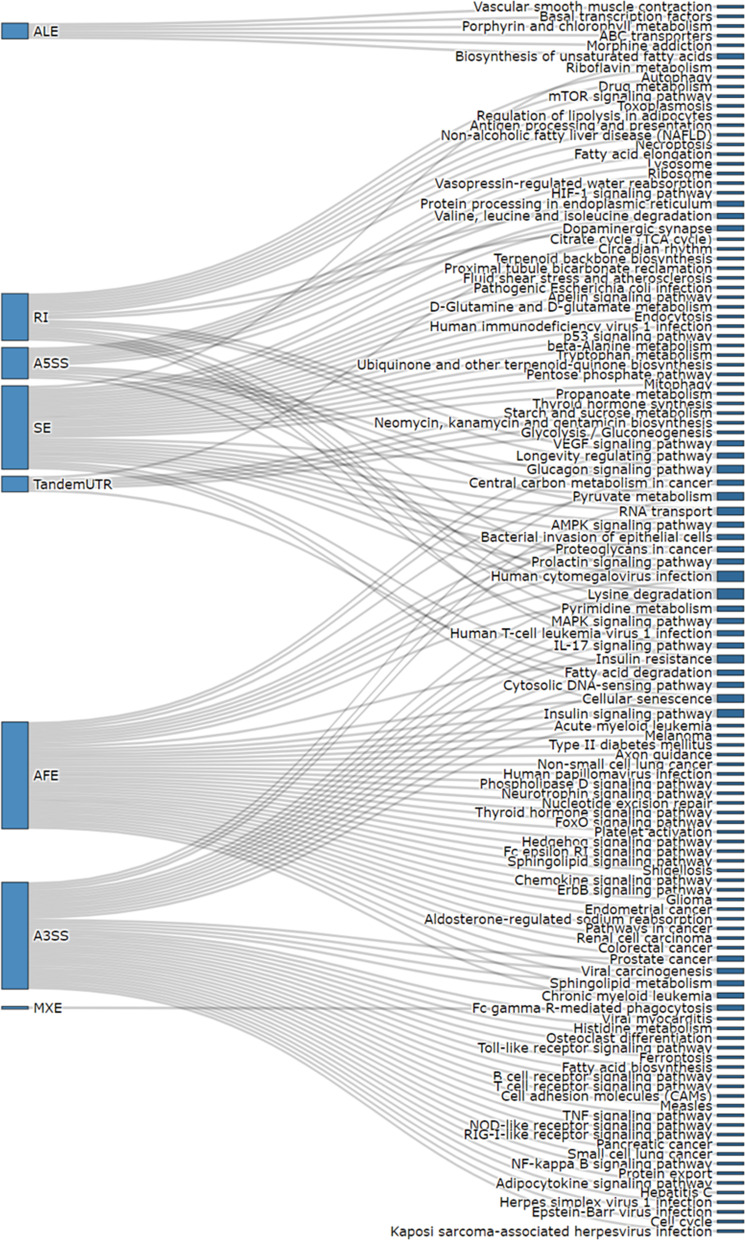
Pathway enrichment analysis. Biological pathways associated with the related alternate splicing events in different splice types

**Fig. 3:**
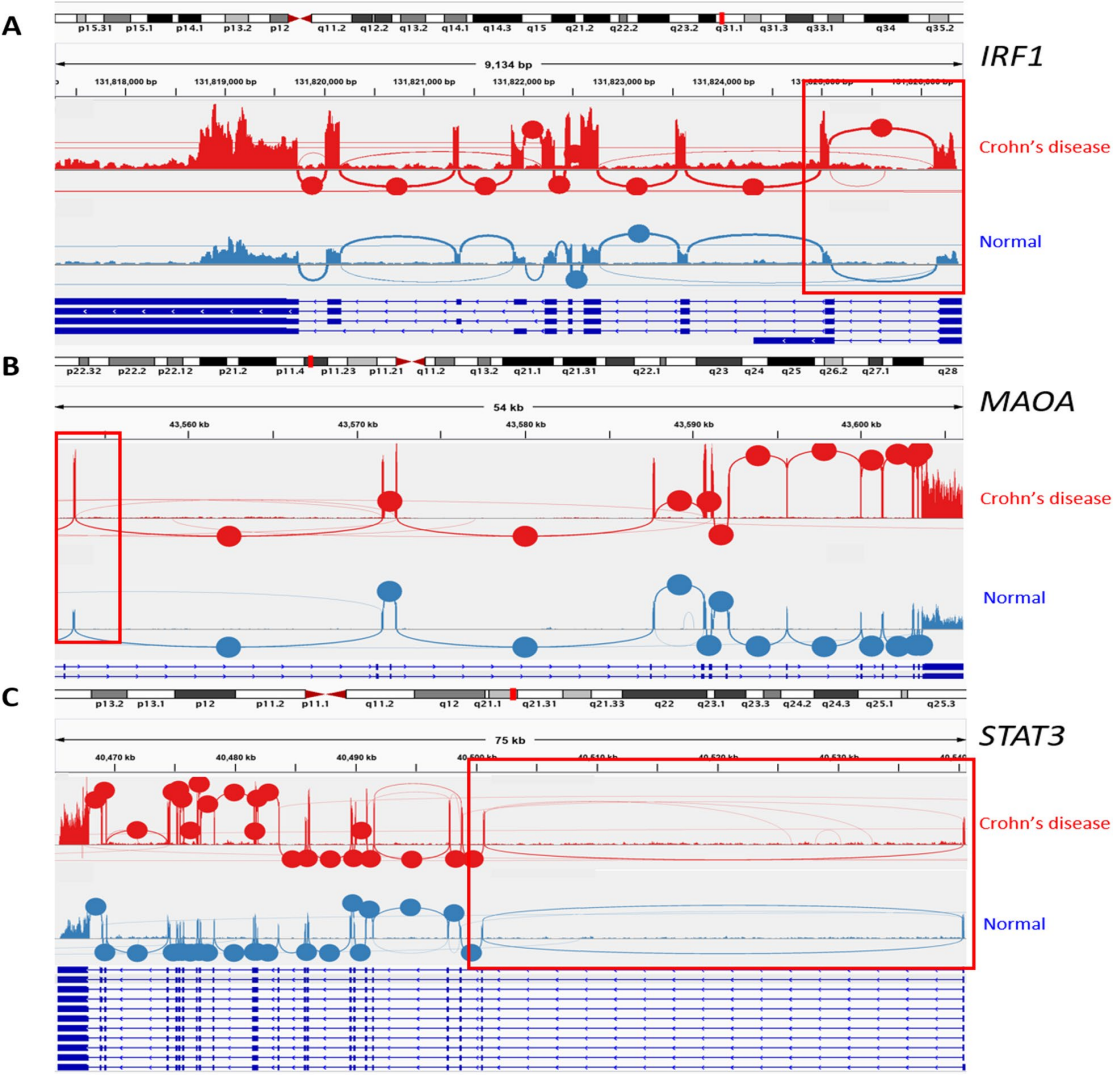
Integrative genomics viewer sashimi plot of three AS related genes (*IRF1*, *MAOA* and *STAT3*) expression and potential splicing patterns (circle = junction coverage, line = potential splicing event)

AS events related to Crohn’s disease contributed to 117 different biological pathways (KEGG pathway) (Fig. 2). Among these significantly enriched pathways, “Lysine degradation,” “Sphingolipid metabolism,” and “Proteoglycans in cancer” were the most enriched terms, with *P* values of 3 × 10^–5^, 3.6 × 10^–4^, and 3.6 × 10^–4^, respectively (Additional file 2: Table S2). Most of the pathways were associated with only one splicing type. However, “Human cytomegalovirus infection” and “Lysin degradation” pathways were affected by the four splicing types. We then combined related AS event pathway analysis results with RNA-seq expression results. We found that eight inflammation-related genes were regulated by CD-related AS events. Among these eight genes, *IRF1*, *STAT3*, and *MAOA* were identified to have the differentially expressed AS events with the lowest *P* values of the Wilcoxon test and the most significant differential expression level between the Crohn’s disease and normal group (Fig. 3a–c). Three genes were observed with SE splicing events (the first exon was skipped; red box in Fig. 3). *STAT3* has also been observed in AFE splicing events. According to the pathway analysis, *IRF1* and *STAT3* are involved in prolactin signaling pathways, which have a multitude of effects related to immunoregulation and protection [27]. Moreover, *MAOA* is related to tryptophan metabolism, which is also highly associated with inflammation, stress response, and microbiome homeostasis [28]. All three genes in the different sample groups showed significantly different RNA expression levels.

### Principal component analysis (PCA) analysis

**Fig. 4:**
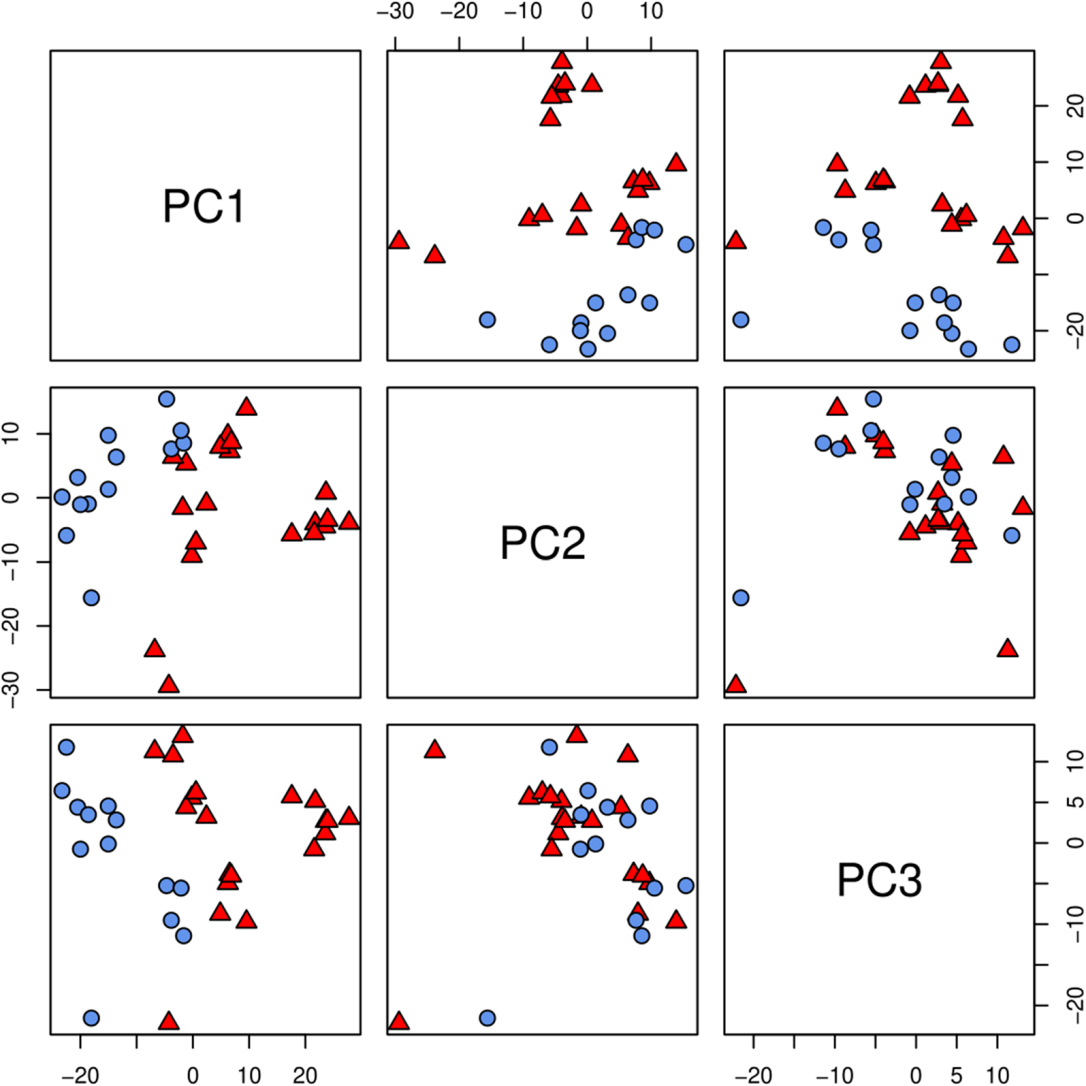
Principal components analysis for the alternative splicing events in 33 samples. Scatterplot pairs of the first three principal components for Percent Spliced In (PSI) values across 33 samples. The red triangles represent CD patients and the blue circle represent normal samples

We summarized 2980 related AS events that occurred across all 33 samples with PSI values generated by MISO. To characterize the AS events between the disease and normal samples, we performed a PCA on the MISO results of 2980 related AS events. PC1–PC3 accounted for 33% of the variance (Fig. 4). Biological differences between CD patients and control group were captured by the first, second, and third principal components.

### Splicing types in the transferability study

To validate AS events that we identified in the GSE66207 datasets, we collected samples and performed RNA-seq on four colon tissues from patients with CD and four controls from the Shengjing Hospital of China Medical University. Between June 2020 and September 2020, a total of four patients diagnosed on the basis of the standard clinical, endoscopic, and histological criteria of CD at the Gastroenterology Department of the Shengjing Hospital of China Medical University were included and served as the validation group. Biopsies were performed during endoscopy. Patients with infectious colitis, indeterminate colitis, Behcet’s disease, intestinal tuberculosis, and colorectal cancer were excluded. Colon biopsies obtained from healthy volunteers during endoscopy served as the control group. Clinical disease activity was assessed by measuring the Crohn’s disease activity index for CD by a physician who was unaware of the patients’ colonoscopy results. This study was approved by the institutional review board of the Shengjing Hospital of China Medical University, and informed consent was obtained from each patient (Table 1).

**Fig. 5:**
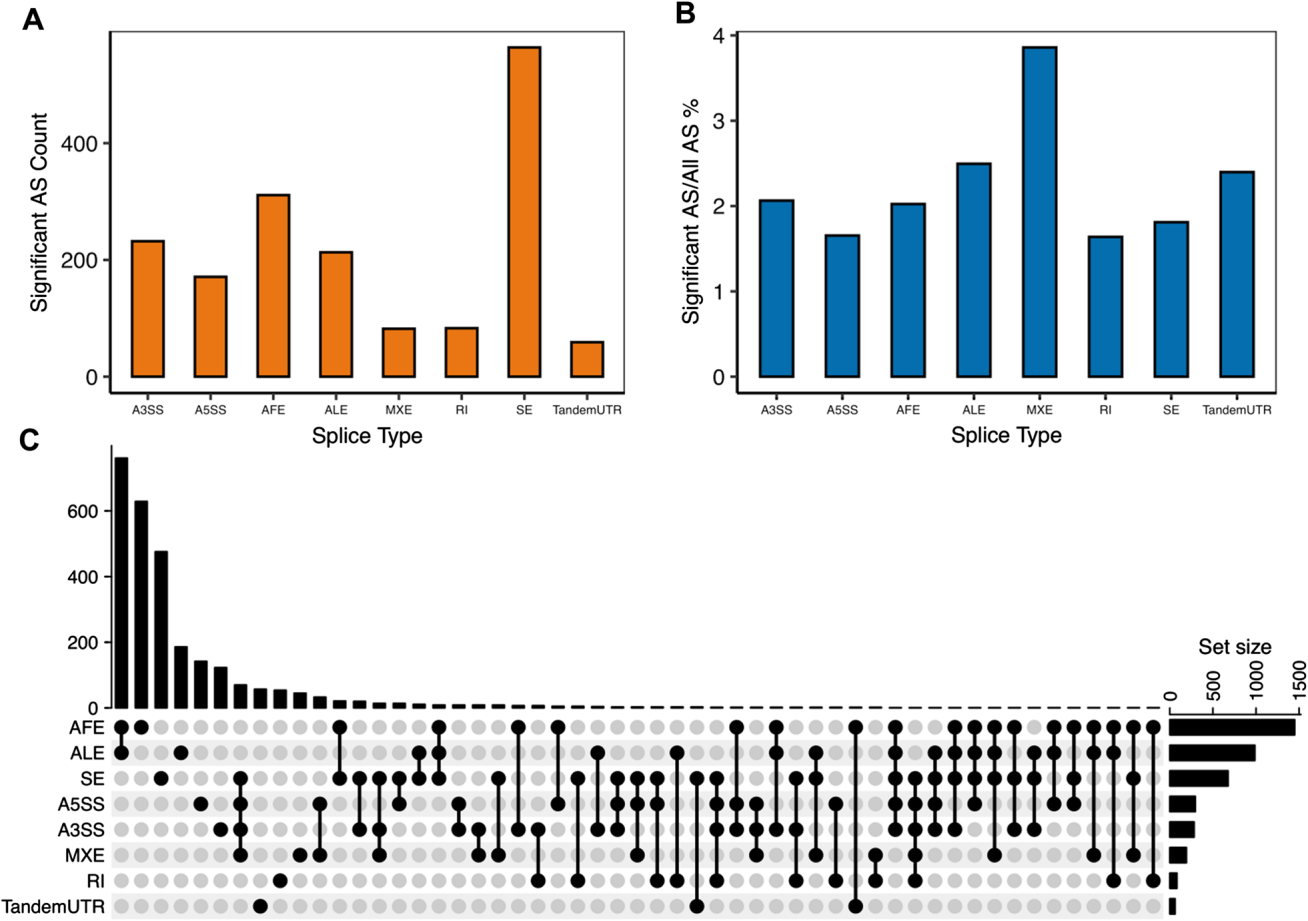
Alternative splicing (AS) identification in Crohn’ disease (CD) patients and normal samples in the transferability study. **a** Significant AS event counts in different AS types between CD patients and normal samples. **b** The percentage of significant AS events over all AS events in different AS types. **c** Distribution of AS types and intersections between gene symbols of 1715 related AS events

Using the same analysis pipeline, we identified 1715 significant AS events (Additional file 3: Table S3) in the validation analysis. Interestingly, these results demonstrated that SE and AFE were still the two most common events, and MXE was the least common, which were similar to results from the public dataset (Fig. 5a). However, the percentage over the total number of all event types was relatively lower than that in the previous study (Fig. 5b). In 1715 significant AS events, AFE and alternative last exon were also identified across multiple genes, which had the most intersections (Fig. 5c).

### Biological process pathway analysis in the transferability study

**Fig. 6:**
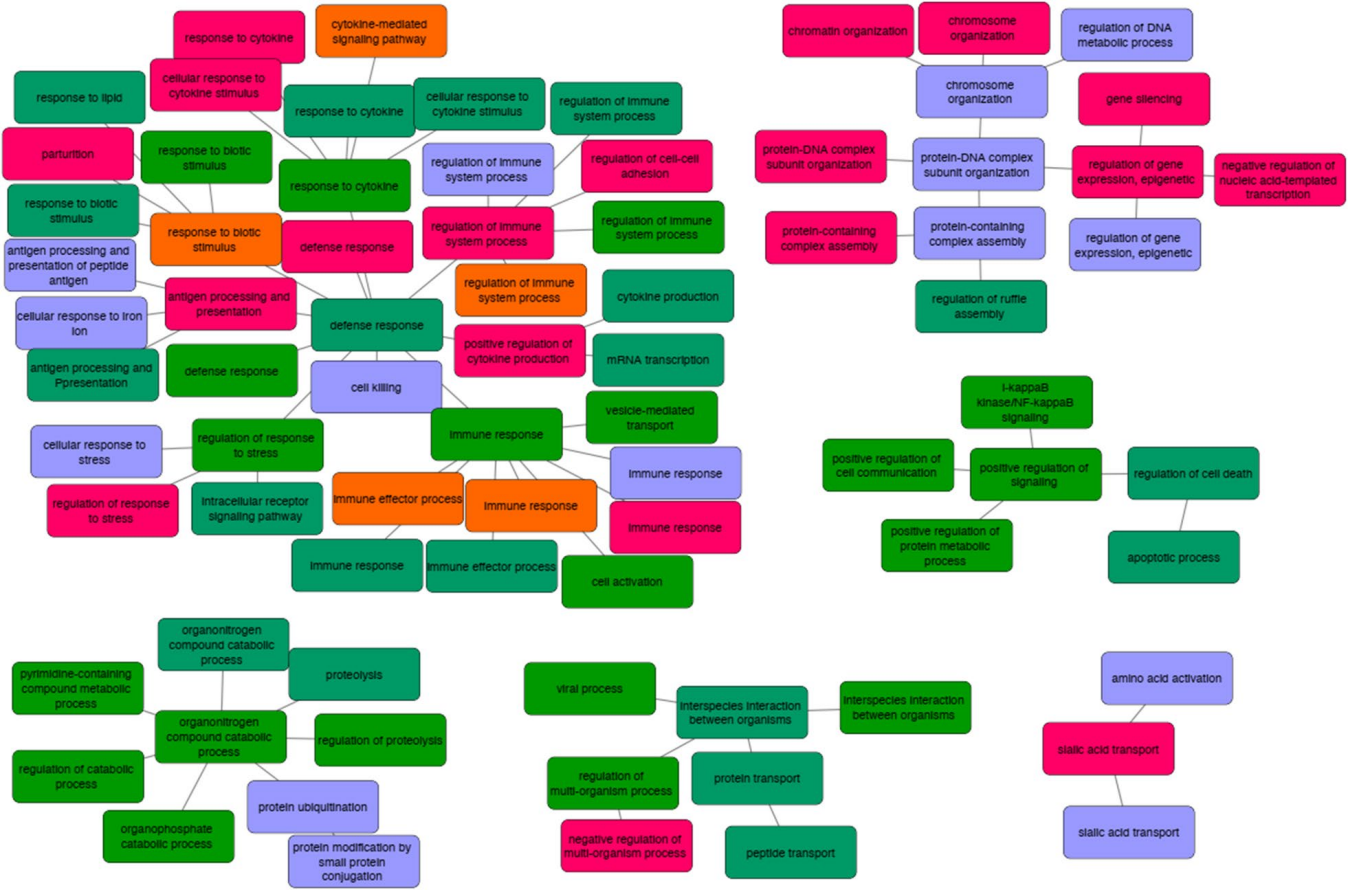
Enriched biological process networks that were identified in our validation study

We performed biological process pathway analysis in a transferability study based on the gene lists of 1731 significant AS events. Since there are less than 200 related genes in MXE, RI, and tandem 3′ UTR event types, we did not identify significant biological processes of these three types of events. In the other five AS-type events, we found that several biological process networks were highly enriched using WebGestalt analysis. Among these significantly enriched networks, “defense response,” “cell killing,” “response to cytokine,” and their related modules were the most enriched clusters (Fig. 6). These results suggest that the potential dysregulation of AS may play important roles in host-microbiome interactions in patients with CD.

### Comparison between the data from public datasets and our transferability study

**Fig. 7:**
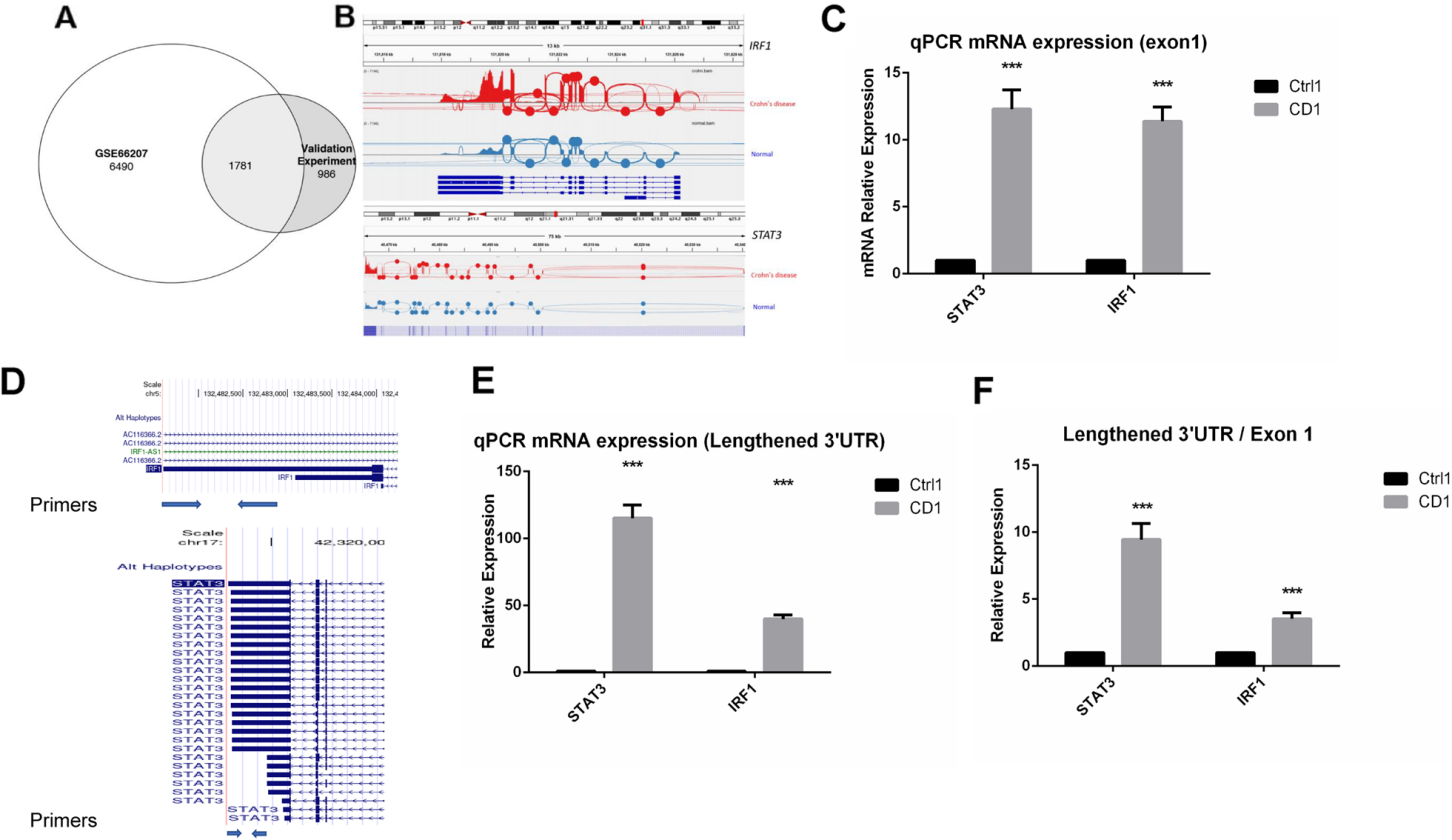
Linkage between results from the public dataset and results from our transferability study. **a** Venn diagram shows the 1781 genes that were identified to include significant AS events in the transferability study that were overlapped with genes of results from GSE66207. **b** IGV Sashimi plot showing *IRF1* and *STAT3* also had significant tandem 3’UTR events, as well as significantly altered expression, in patients with CD compared to the controls. **c** Quantitative real-time PCR (RT-qPCR) analysis showing that the exon1 mRNA expression of the *STAT3* and *IRF1* were both significantly upregulated in the CD group. ***Student’s t-test *P* value < 0.001; bar = SEM. **d** Scheme shows how primers were designed to target the lengthened 3’UTR of the *STAT3* and *IRF1* genes in the CD patients. **e** RT- qPCR analysis results show that the expression of the lengthened 3’UTR region of *STAT3* and *IRF1* were significantly upregulated in the CD samples, matching the results of **b**. ***Student’s t-test *P* value < 0.001; bar = SEM. **f** Normalized results of the lengthened 3’UTR/exon 1 expression of the *STAT3* and *IRF1* gene. ***Student’s t-test *P* value < 0.001; bar = SEM

Finally, we compared results from the analysis of the public dataset and results from the analysis of our own cohort. In terms of genes that had significant AS events, we identified 64% genes that were identified to include significant AS events in the transferability study and overlapped with genes from the results we found in the public dataset (Fig. 7a). Interestingly, *IRF1* and *STAT3* were still identified to have significant tandem 3’UTR events and showed significantly altered expression at the mRNA level in the patients (Fig. 7b). To validate these findings, we performed RT-qPCR assays on eight samples (4 CD vs. 4 control). We initially validated the upregulation of *STAT3* and *IRF1* expression in the CD samples (Fig. 7c), which is consistent with previous findings [29, 30]. We also performed RT-qPCR assays specifically targeting different uses of the 3’UTR regions of these two genes. Interestingly, the expression levels of the lengthened 3’UTR were significantly increased in the CD samples, compared to those in the control (Fig. 7d, e). Even after normalization to the overall mRNA expression (Exon 1), the lengthened 3’UTR expression was still profoundly upregulated in the CD samples (Fig. 7f). These results further validated our findings of significant AS in crucial genes related to CD, indicating the potential role of AS in regulating CD-related gene expression, thus contributing to the etiology of the disease. Finally, we collected 404 splicing factors from a large splicing factor database for expression analysis [31]. We excluded 34 factors that were barely expressed in the RNA-seq data (fragments per kilobase of transcript per million mapped reads (FPKM) < 1). As a result, we identified five splicing factor genes that were significantly differentially expressed (*P* value < 0.05) in both datasets (Table 2). We also noticed that these splicing factors had the same trend in terms of expression level alteration. We speculate that these splicing factors may be important in regulating AS events in patients with CD. In conclusion, these results suggest a strong similarity between our transferability study and the public dataset. We also speculate the difference from these analyses may be due to different genetic backgrounds of these samples, as the first cohort was from the University of North Carolina (presumably a cohort of mixed ethnicity) and the validation cohort consisted of only Han Chinese. The average age of the first cohort was 45 years old, and the average age of our cohort was 21 years old, indicating a significant difference in age between the two populations. However, the sex ratio between the two cohorts was similar (1:1 vs. 0.7:1). These factors could also potentially affect AS results.

**Table 2:** Transferability study RNA-seq splicing factor raw count fold change of 8 samples

Gene symbol	Description	GSE66207-Log2Foldchange CD versus control	Validation experiment- Log2Foldchange CD versus control
HSPA1A	Heat shock protein family A (Hsp70) Member 1A	2.4950086	1.73285
RNF213	Ring finger protein 213	1.5825733	3.760895
MOV10	Mov10RISC complex RNA helicase	1.5125286	1.304929
HSPA5	Heat shock protein family A (Hsp70) Member 5	1.3313641	3.431851
LSM10	LSM10, U7 small nuclear RNA associated	1.2935081	1.631783

## Discussion

Dysregulation of pre-mRNA splicing can cause human disease, and an increasing number of studies have shown that targeting AS could lead to the development of novel therapeutics [32]. To date, accumulated data have already highlighted crucial roles of AS in many human diseases, including cancer, neurodegenerative disorders, and immune and infectious diseases [33–35]. Herein, we present a comprehensive analysis of AS events in CD using a well-established public dataset and a validation dataset from sequencing a Chinese cohort. In our study, we found that SE was the most significantly enriched AS event in patients with CD. This suggests that transcriptome of CD patients is much more complex and chaotic compared to that of healthy controls, due to a more sophisticated mRNA isoform system. The PCA plot also demonstrated that even if only different AS events were used as principal components, CD and control groups were still distinguishable, suggesting that the mRNA AS event itself, other than the expression level, could be a solid comprehensive signature for CD patients. All these results proved our hypothesis that dysregulated AS events may contribute to the etiology of CD.

Among AS events related to significantly enriched pathways, the lysine degradation” pathway was the most enriched pathway. *EZH1* and *EZH2* are two genes involved in this pathway, which have been previously linked to the progression of IBD [36, 37]. “Sphingolipids metabolism” is the most significantly enriched pathway in A3SS events. Interestingly, sphingolipids have recently been recognized as mediators of inflammation and are potential therapeutic targets for IBDs [38]. Our results suggest that the metabolism of sphingolipids in CD patients may differ from that in healthy individuals due to AS, resulting in a dysregulated sphingolipid pool. Moreover, the three most dysregulated genes in inflammatory response-related pathways were previously linked to IBD. *IRF1*, as an interferon regulatory factor, has been correlated with the CD activity index and CD endoscopic index of severity [39]. In a cohort study, *IRF1* showed a 72% increase in gene expression in patients with CD compared to that in controls [40]. If *STAT3*, as a central component in immune response signal transduction, has a gain-of-function mutation, it will cause multi-organ early auto-immune diseases [41]. Notably, *STAT3* has also been indicated as a crucial target for treating IBDs, although activation of *STAT3* is likely to occur in both innate and acquired cell types [29]. *MAOA*, as a monoamine oxidase, is not directly associated with IBDs and is highly related to the induction of oxidative stress in obese people suffering from chronic inflammation [42]. Recently, dysregulation of *MAOA* has also been linked to specific microbe alterations [43]. In conclusion, both top AS-related significantly enriched pathways and significantly dysregulated genes were highly related to IBD and CD. Our results provide new insights into the role of post-transcriptional regulation in IBD by regulating these key pathways and genes.

Although our study provided a comprehensive AS analysis of colon tissues from CD patients and also identified nearly 3000 unique AS events in CD patients, our study still has certain limitations. First, although we validated the AS events of *STAT3* and *IRF1* genes, the experimental validation (protein level) of discovered AS events in many other genes is still lacking. In fact, recent updates on AS effect prediction algorithm have allowed us to predict the function alteration of the targeted proteins. 68 AS events were predicted to alter the downstream protein function or expression by using ASpedia software. These affected AS events include 10 nonsense-mediated decay (NMD) related events, 22 protein domain related alterations, 57 posttranslational modification related events (Additional file 4: Table S4). However, whether these AS events affect protein diversity or not still requires experimental validation. Validation studies should examine protein levels of these AS-regulated genes and detect downstream effects on certain signal transduction pathways. Finally, even if we have detected several splicing factors that were dysregulated in CD patients, an RNA immunoprecipitation sequencing or cross-linking immunoprecipitation study should be performed to further detect responsible splicing factors and RNA-binding proteins for CD-related AS events. Nonetheless, our study demonstrated a new potential pathophysiology of CDs, which is regulated by AS. Our study also presented the first AS landscape in patients with CD and suggested that drugs that target AS-related genes and splicing factors should be considered for further screening of patients with CD.

## Conclusion

Our study presented a landscape of AS in CD and provided the first AS-related transcriptional analysis in a Chinese cohort. Integrated analysis of the two datasets revealed that AS may play a crucial role in determining the pathogenesis of CD.

## Supplementary Information

**Additional file 1: Table S1**. Table for discovered significant AS events in the 20 CD patients from public dataset

**Additional file 2: Table S2**. Table for KEGG pathway enrichment of AS events in CD patients

**Additional file 3: Table S3**. Table for discovered significant AS events in our cohort

**Additional file 4: Table S4**. Table for AS related effects on protein function/diversity for the public dataset analysis

## Data Availability

RNA-seq data for 20 CD AS anakysis was obtained from NCBI GEO dataset (GSE66207). “The datasets generated and/or analysed during the current study are available in the NCBI_GEO repository, [PERSISTENT WEB LINK OR ACCESSION NUMBER TO DATASETS]”.

## Abbreviations

CD: Crohn’s disease
AS: Alternative splicing
IBD: Inflammatory bowel disease
miRNAs: Micro-RNA
KEGG: Kyoto encyclopedia of genes and genomes
FDR: False discovery rate
3’ UTR: Three prime untranslated region
*IRF1*: Inflammation-related responses. Interferon regulatory factor 1
*STAT3*: Signal transducer and activator of transcription 3
*MAOA*: Monoamine oxidase A
